# Recruitment of regulatory T cells is correlated with hypoxia-induced CXCR4 expression, and is associated with poor prognosis in basal-like breast cancers

**DOI:** 10.1186/bcr2869

**Published:** 2011-04-26

**Authors:** Max Yan, Nicholas Jene, David Byrne, Ewan KA Millar, Sandra A O'Toole, Catriona M McNeil, Gaynor J Bates, Adrian L Harris, Alison H Banham, Robert L Sutherland, Stephen B Fox

**Affiliations:** 1 Department of Pathology, Peter MacCallum Cancer Centre, St Andrews Place, East Melbourne, Melbourne, VIC 3002, Australia; 2 Cancer Research Program, Garvan Institute of Medical Research, 384 Victoria Street, Darlinghurst, Sydney, NSW 2010, Australia; 3 School of Medical Sciences, University of New South Wales, High Street, Kensington, Sydney, NSW 2052, Australia; 4 South East Area Laboratory Services, St George Hospital, South Street, Kogarah, Sydney, 2217, Australia; 5 School of Medicine, University of Western Sydney, Narellan Road, Campbelltown, Sydney, NSW 2560, Australia; 6 Department of Tissue Pathology, Royal Prince Alfred Hospital, Missenden Road, Camperdown, Sydney, NSW 2010, Australia; 7 Department of Medical Oncology, Royal Prince Alfred Hospital, Missenden Road, Camperdown, Sydney, NSW 2010 Australia; 8 Nuffield Department of Clinical Laboratory Sciences and Medical Oncology, University of Oxford, Henry Wellcome Building for Molecular Physiology, Old Road Campus, Headington, Oxford, OX3 7BN, UK; 9 Weatherall Institute of Molecular Medicine, University of Oxford, John Radcliffe Hospital, Oxford, OX3 9DS, UK

## Abstract

**Introduction:**

Basal-like breast cancers behave more aggressively despite the presence of a dense lymphoid infiltrate. We hypothesised that immune suppression in this subtype may be due to T regulatory cells (Treg) recruitment driven by hypoxia-induced up-regulation of CXCR4 in Treg.

**Methods:**

Immunoperoxidase staining for FOXP3 and CXCL12 was performed on tissue microarrays from 491 breast cancers. The hypoxia-associated marker carbonic anhydrase IX (CA9) and double FOXP3/CXCR4 staining were performed on sections from a subset of these cancers including 10 basal-like and 11 luminal cancers matched for tumour grade.

**Results:**

High Treg infiltration correlated with tumour CXCL12 positivity (OR 1.89, 95% CI 1.22 to 2.94, *P *= 0.004) and basal phenotype (OR 3.14, 95% CI 1.08 to 9.17, *P *= 0.004) in univariate and multivariate analyses. CXCL12 positivity correlated with improved survival (*P *= 0.005), whereas high Treg correlated with shorter survival for all breast cancers (*P *= 0.001), luminal cancers (*P *< 0.001) and basal-like cancers (*P *= 0.040) that were confirmed in a multivariate analysis (OR 1.61, 95% CI 1.02 to 2.53, *P *= 0.042). In patients treated with hormone therapy, high Treg were associated with a shorter survival in a multivariate analysis (OR 1.78, 95% CI 1.01 to 3.15, *P *= 0.040). There was a tendency for luminal cancers to show CXCL12 expression (102/138, 74%) compared to basal-like cancers (16/27, 59%), which verged on statistical significance (*P *= 0.050). Up-regulation of CXCR4 in Treg correlated with the basal-like phenotype (*P *= 0.029) and tumour hypoxia, as indicated by CA9 expression (*P *= 0.049).

**Conclusions:**

Our data show that in the setting of hypoxia and CXCR4 up-regulation in Treg, CXCL12 expression may have the negative consequence of enhancing Treg recruitment and suppressing the anti-tumour immune response.

## Introduction

Cancer is rarely suppressed by the host immune response since tumour cells acquire immune tolerance. The failure of an anti-cancer immune response may be due to a specific subpopulation of regulatory T cells (Treg) [1]. Treg down-regulate the activation and expansion of self-reactive lymphocytes [2], and are crucial for the repression of autoimmune disorders and transplant rejection [3,4]. Although the role of Treg in cancer has not been fully elucidated, these cells are likely to be responsible for maintaining the self-tolerance that may hinder the generation and activity of anti-tumour reactive T cells [2]. This is supported by observations that depletion of Treg [1,5,6] and transforming growth β secreted by Treg [7,8] correlate with an enhanced immune response to cancer vaccines. Recently we and others have demonstrated that tumour infiltration by Treg, independent of other lymphoid populations, is associated with a reduced survival in breast and other cancers [9-13].

Breast cancers are heterogeneous and one recognised subgroup, basal-like breast cancers, derive their name from the characteristic expression of basal cytokeratins (CK) 5, 14 and 17 [14,15]. These tumours account for up to 15% of all invasive breast cancers [16], and are frequently observed in patients with BRCA1-related cancers [17]. Despite the presence of a dense lymphoid infiltrate on histology, which is suggestive of an anti-tumour immune response [17], they are associated with a more aggressive clinical course characterised by shorter survival and a higher risk of metastasis [17]. We hypothesize that this is due, in part, to suppression of the immune response by Treg.

In non-neoplastic tissues, Treg are recruited by chemokines such as CXCL12 secreted by bone marrow, lymph node and inflammatory cells [18], a mechanism that is replicated in tumours through chemokine secretion by neoplastic cells [18]. Thus CXCL12, which binds to its cognate receptor CXCR4 expressed by Treg, has been implicated in the recruitment of Treg in a number of tumours including ovarian cancer [19], adenocarcinoma of the lung [20], malignant mesothelioma [21], and the myelodysplastic syndromes [22]. CXCR4 expression is induced under hypoxic stress via activation of the HIF pathway in a number of cell types including B lymphocytes [23], tumour associated monocytes and endothelial cells [24], microglia [25], multipotent stem cells, stromal cells [26,27], cardiac monocytes and fibroblasts [28]. Furthermore, the HIF pathway enhances the immunosuppressive activity of Treg by promoting the expression of their lineage transcriptional regulator FOXP3 [29]. Given the role of hypoxia in T cell activation [30,31], and also specifically in Treg [29], we hypothesised that Treg recruitment is dependent on both CXCL12 production by tumour cells and hypoxia-induced CXCR4 expression in Treg. We further hypothesize that since basal-like tumours have an enhanced hypoxic drive [32] this mechanism may be prominent in basal-like breast cancer.

We, therefore, investigated CXCR4 expression in Treg, together with the expression of CA9 and CXCL12 in basal-like and other subtypes of breast cancers. The significance of this project lies in the rational design of tumour vaccine approaches or blocking antibodies [33]. Therapies targeting Treg are entering clinical trials [34,35]; therefore, it is important to quantify Treg numbers and to assess factors that may affect their recruitment to the tumour microenvironment. Thus, should findings suggest that hypoxia driven recruitment of Treg via the CXCL12/CXCR4 axis plays a significant role in basal-like tumours, therapies targeting CXCL12/CXCR4 and HIF pathways, in addition to targeting Treg may be beneficial for this subset of breast cancers that are less likely to respond to conventional therapies.

## Materials and methods

### Patient characteristics

The flow of patients through the study as per the REporting recommendations for tumour MARKer prognostic studies (REMARK) criteria [36] is as follows. Six hundred and twenty-one invasive breast carcinomas, characterised in a previous study [32], were retrospectively collected from the John Radcliffe Hospital, Oxford, UK and from the Garvan Institute of Medical Research, Sydney, Australia. Of the 621 tumours, 594 tumours had tissue available for tissue microarray (TMA) construction, of which 491 tumours were available for FOXP3 staining due to core drop-out. A subset of these tumours (254 cases) was also stained for CXCL12. Arrays contained duplicate cores (1 mm cores). FOXP3/CXCR4 double staining and CA9 staining were performed on whole stained sections of 10 grade 3 basal-like and 11 grade 3 luminal cancers from the Peter MacCallum Cancer Centre. A waiver for informed consent, for the use of archival material, has been obtained as part of Ethics Committee approvals (JR C02.216, HREC SVH H94/080 and PMCC 09/36). All patients had operable breast carcinomas and were not diagnosed with metastatic disease at the time of presentation. Median age of patients included in this study was 55 years (range 24 to 87 years). Median tumour size was 20 mm and the median tumour grade was 2. Forty-four percent of patients had nodal disease. Sixty-eight percent of tumours were ER positive and 16% were HER2 positive. A total of 198 patients (31.9%) received adjuvant chemotherapy (cyclophosphamide, methotrexate and 5-fluorouracil (CMF) or adriamycin and cyclophosphamide (AC)). Adjuvant tamoxifen was given to 222 (35.7%) patients. Patients were followed-up for a median period of 131.9 months. During this time, 137 patients developed recurrence (30.0%) and 99 deaths (21.7%) were considered breast-cancer related.

### Immunohistochemistry and scoring

Paraffin embedded tissues were dewaxed. For FOXP3 and CA9 staining, antigen retrieval was performed by microwaving in 50 mmol/L Tris/2 mmol/L EDTA (pH 9.0). Labelling was performed using the mouse monocolonal antibodies 236A/E7 (FOXP3, Abcam, Cambridge, UK) at 10 μg/mL [11] and M75 (CA9) at 1:100 [32]. For CXCL12 immunohistochemistry, antigen retrieval was performed using the DAKO PT Link retrieval system (Glostrup, Denmark) at high pH for 20 minute at 100°C. Labeling was performed using mouse CXCL12 antibody (R&D Systems, Minneapolis, MN, USA, MAB350) [37] diluted to 8 μg/mL. Labeling was detected using the Dako Envision System. The stained arrays were counterstained with haematoxylin. Positive and negative staining controls for both antibodies were carried out in parallel using tonsillar tissue.

Double immunohistochemical staining on whole sections for FOXP3 and CXCR4 was performed using the Ventana Benchmark^® ^ULTRA system (Tucson, Arizona, USA). Antigen retrieval was performed using Ventana ULTRA Cell Conditioning 1 (95°C for 36 minutes). Sections were incubated with FOXP3 antibody (10 μg/mL) for 20 minutes (room temperature), followed by visualization with Ventana Ultraview Universal DAB. This was followed by incubation with polyclonal rabbit CXCR4 antibody (Sigma, St Louis, MO, USA, C3116) at 4 μg/mL [38] for 16 minutes (room temperature) and visualization with Ventana Ultraview Alkaline Phosphatase Red Detection Kit. Centroblasts and centrocytes within lymph node germinal centres were used as positive and negative controls respectively [39].

**Figure 1:**
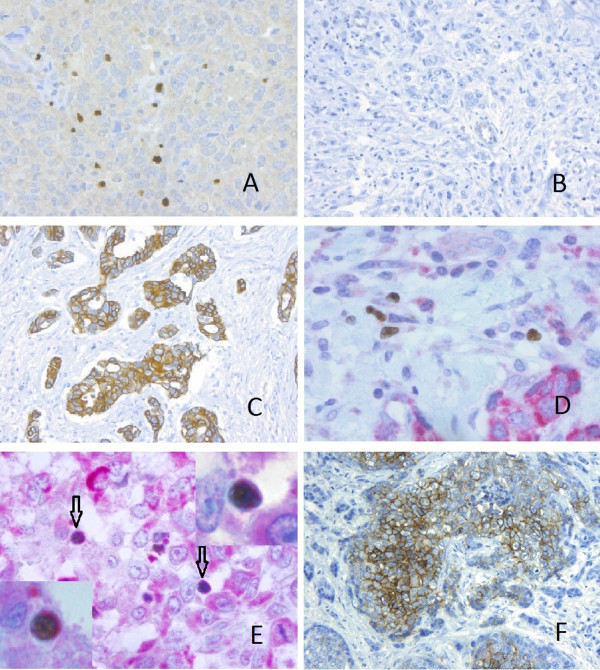
**Immunohistochemistry for FOXP3, CXCL12, CXCR4 and CA9**. Immunohistochemical staining for **(A) **FOXP3 positive tumour infiltrating Treg (x20), **(B) **Tumour cells negative for CXCL12 (x10), **(C) **Tumour cells showing strong positivity for CXCL12 (x10). **(D, E) **Double immunohistochemical staining for FOXP3 (brown, nuclear) and CXCR4 (red, cytoplasmic). **(D) **CXCR4 negative Treg (x40), **(E) **CXCR4 positive Treg (arrows) (x40), Inset (x100 oil immersion), **(F) **Tumour cells with positive CA9 staining.

Cores were available for 456 tumours to be classified into four intrinsic subgroups, based on ER, HER2 *in situ *hybridization, EGFR and CK5/6 staining, as per Nielson *et al. *[16] and Millar *et al. *[40]: basal-like group (ER negative, HER2 negative, CK5/6 and/or EGFR positive), luminal group (ER positive, HER2 negative), HER2 group (HER2 positive) and a negative group (ER, HER2, CK5/6 and EGFR negative). Absolute numbers of FOXP3-positive, Treg lymphocytes in assessable 1-mm diameter invasive tumour cores were counted manually (Figure 1a). A cut-off of 15 Treg per core was used to divide the tumours into two groups (as previously defined by Bates *et al. *[11]) The level of staining for CXCL12 was scored with respect to the intensity and percentage of staining in the cytoplasm. The scoring system for intensity was: 0 = no staining, 1 = weak staining, 2 = moderate staining, 3 = strong staining. The percentage of tumour cells stained in the given core scored as: 0% = 0; 1 to 10% = 1; 11 to 50% = 2; 51 to 80% = 3; 81 to 100% = 4. The CXCL12 scores for both staining intensity and the percentage of positive tumour cells were added together to give a maximum score of 7. A CXCL12 cut-off score of 7 was used to divide the tumours into approximately two equal groups (Figures 1b, c). For FOXP3/CXCR4 double staining, the number of Treg co-expressing FOXP3/CXCR4 was enumerated from four (x40, 0.55 mm diameter) high power fields of lymphoid rich infiltrate (approximately equal to 0.95 mm^2^) within the tumour (Figures 1d, e). For CA9 staining, positive expression was defined as the presence of strong membranous staining in ≥10% of tumour cells (Figure 1f) [32,41].

### Statistical methods

Group comparisons were made for non-thresholded data using unpaired t test, and for thresholded data using the chi square test. Kaplan-Meier survival curves were plotted using breast cancer related death as the endpoint and compared using a log rank test. Binary logistic regression was used for multivariate analyses and the Cox proportional hazard regression model was used to identify independent prognostic factors for overall survival. Analyses were performed with SPSS 16.0 (SPSS Inc., 233 South Wacker Drive, Chicago, IL, 60606, USA). A two-tailed *P-*value test was used in all analyses and a *P-*value of less than 0.05 was considered statistically significant.

## Results

### Tumour infiltration by Treg correlates with tumour CXCL12 expression and basal phenotype

The number of Treg was correlated with clinicopathological parameters (Table 1). In a univariate analysis, tumours with high Treg (defined as ≥15 Treg per core) were significantly more frequently grade 3 tumours (115/224, 51%), compared with tumours with low Treg (80/259, 31%) (*P *< 0.001). Compared to low Treg tumours, high Treg tumours also significantly correlated with negative ER status (73/262 (28%) and 81/216 (38%) ER negative respectively, *P *= 0.025), positive HER2 status (7/245 (3%) and 20/206 (10%) HER2 positive respectively, *P *= 0.002) and positive CXCL12 expression (74/125 (59%) and 83/110 (75%), *P *= 0.008). No correlation was observed between Treg and nodal status, (*P *= 0.062), endocrine therapy (*P *= 0.255) and chemotherapy (P = 0.148). Basal-like cancers were more likely to have high Treg (36/55, 75%, median 25.46) compared with luminal cancers (99/258, 38%, median 7.64) (*P *< 0.001, Table 2).

**Table 1:** Correlation of FOXP3 Treg with clinicopathological parameters and CXCL12 expression

	Treg < 15	Treg ≥15	*P-*value
**Tumour size (mm)**			
Median	21.2	23.8	0.571
**Tumour grade**			< 0.001
1	56 (22%)	25 (11%)	
2	123 (47%)	84 (38%)	
3	80 (31%)	115 (51%)	
**Nodal status**			0.062
Negative	154 (59%)	109 (51%)	
Positive	107 (41%)	107 (49%)	
**ER**			0.025
Negative	73 (28%)	81 (38%)	
Positive	189 (72%)	135 (62%)	
**HER2**			0.002
Negative	238 (97%)	186 (90%)	
Positive	7 (3%)	20 (10%)	
**CXCL12**			0.008
Negative	51 (41%)	27 (25%)	
Positive	74 (59%)	83 (75%)	
**Endocrine Rx**			0.255
Negative	99 (37%)	72 (32%)	
Positive	167 (63%)	151 (68%)	
**Chemotherapy**			0.148
Negative	168 (64%)	127 (57%)	
Positive	96 (35%)	95 (43%)	

ER, estrogen receptor; Rx, therapy.

**Table 2:** Correlation analysis of intrinsic subtypes with clinicopathological parameters, Treg and CXCL12 expression (n = 456)

	Luminal (n = 289)	HER2 (n = 75)	Basal-like (n = 62)	Negative (n = 30)	*P-v*alue
**Patient age**					
Median (years)	56.0	55.2	54.0	52.7	0.64
**Tumour size**					
Median	18.0	22.0	22.5	20.5	0.40
**Tumour grade**					< 0.001
1	71 (25%)	4 (5%)	2 (3%)	4 (13%)	
2	156 (55%)	16 (22%)	12 (20%)	12 (40%)	
3	58 (20%)	54 (73%)	47 (77%)	14 (47%)	
**Nodal status**					0.75
Negative	162 (56%)	40 (54%)	34 (55%)	17 (57%)	
Positive	125 (44%)	34 (46%)	28 (45%)	13 (43%)	
**Treg**					< 0.001
< 15	159 (62%)	28 (39%)	19 (25%)	23 (92%)	
≥15	99 (38%)	43 (61%)	36 (75%)	2 (8%)	
median	7.64	33.54	25.46	0.83	
**CXCL12**					0.050
Negative	36 (26%)	22 (47%)	11 (41%)	6 (35%)	
Positive	102 (74%)	25 (53%)	16 (59%)	11 (65%)	

Treg, regulatory T cells.

In a multivariate analysis using the binary logistic regression model, high Treg significantly correlated with basal-like phenotype (OR 3.14, 95% CI 1.08 to 9.17, *P *= 0.004) and CXCL12 expression (OR 1.89, 95% CI 1.22 to 2.94, *P *= 0.004), but not tumour grade (OR 1.43, 95% CI 0.88 to 2.32, *P *= 0.151, Table 3).

**Table 3:** Multivariate analysis, correlation of high Treg (≥15) with tumour size, grade, CXCL12 and tumour type

	Odds ratio	95% CI	*P-*value
Tumour size	0.56	0.27 to 1.14	0.560
Grade	1.43	0.88 to 2.32	0.151
CXCL12	1.89	1.22 to 2.94	0.004
Tumour type			
Luminal (baseline)	1.00	-	-
Basal-like	3.14	1.08 to 9.17	0.004

n = 165

### High Treg infiltration is associated with reduced breast cancer-specific survival in luminal and basal-like cancers

**Figure 2:**
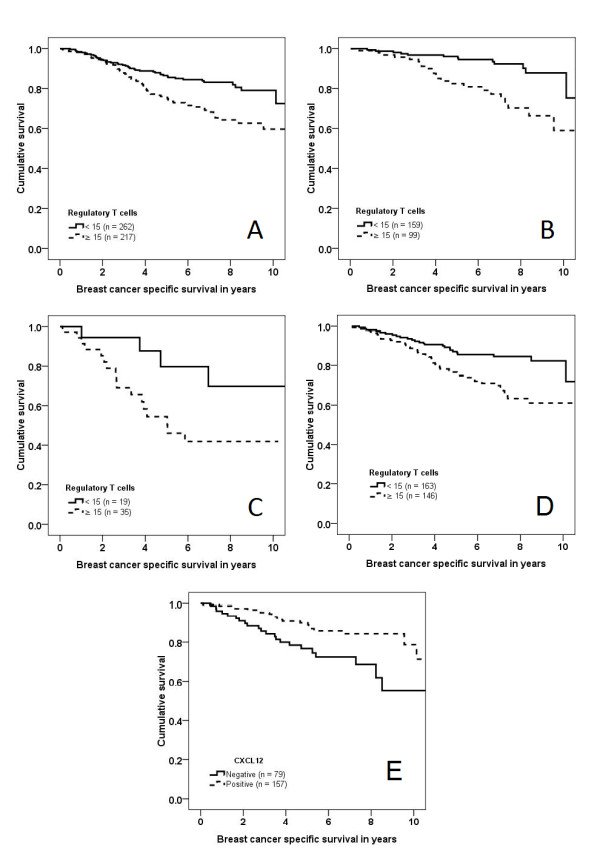
**Kaplan Meier curves, breast cancer specific survival**. Kaplan Meier curves, breast cancer specific survival, **(A) **all cancers stratified by Treg infiltration (n = 479, *P *= 0.001), **(B) **luminal cancers stratified by Treg infiltration (n = 258, *P *< 0.001), **(C) **basal-like cancers stratified by Treg infiltration (n = 54, *P *= 0.040), **(D) **patients treated with hormone therapy (n = 309, *P *= 0.001), **(E) **all cancers stratified by CXCL12 expression (n = 236, *P *= 0.005),

High tumour infiltration by Treg (≥15) was significantly associated with reduced breast cancer specific survival (*P *= 0.001), with divergence of the survival curves occurring two years after initial surgical treatment (Figure 2a). The association of high Treg infiltration with poorer survival in all tumours was confirmed in a multivariate analysis including age, lymph node status, grade, size, ER, HER2 status, hormone therapy and chemotherapy (OR 1.62, 95% CI 1.02 to 2.55, *P *= 0.040) (Supplementary Table S1a in Additional file 1).

Breast cancer-specific survival based on Treg infiltration was analysed for each intrinsic subtype. High Treg infiltration correlated with poorer survival in luminal (*P *< 0.001) (Figure 2b) and basal-like cancers (*P *= 0.040) (Figure 2c), but not in HER2 (*P *= 0.255) or null type cancers (*P *= 0.128).

Treg infiltration was associated with reduced breast cancer-specific survival in patients treated with hormone therapy (*P *= 0.001) (Figure 2d). This was confirmed in a multivariate analysis where high Treg were an independent predictor of a poor response to hormone therapy (OR 1.78, 95% CI 1.01 to 3.15, *P *= 0.040) (Supplementary Table S1b in Additional file 1). No differences in survival were observed for patients treated with chemotherapy when stratified by Treg infiltration (*P *= 0.565)

### CXCL12 expression does not differ between the different intrinsic subtypes, and is associated with longer survival

Although a smaller proportion of basal-like (16/27, 59%) and HER2 tumours (25/47, 53%) was positive for CXCL12 expression compared with luminal tumours (102/138, 74%), this did not reach statistical significance (*P *= 0.050) (Table 2). There was no correlation between CXCL12 expression and tumour size, grade, lymph node status, ER, endocrine treatment and chemotherapy (all *P *> 0.05) (Supplementary Table S2 in Additional file 1). On a log rank test, positive CXCL12 expression correlated with longer breast cancer-specific survival (*P *= 0.024) (Figure 2e).

### Treg infiltration correlates with tumour hypoxia

Carbonic anhydrase IX (CA9) is a transmembrane protein involved in maintaining a low pericellular pH through the conversion of carbon dioxide to carbonic acid [42]. Its expression has been shown to correlate with hypoxia as measured by Eppendorf microelectrode [43] and the distribution of pimonidazole (a chemical marker of hypoxia) [42,44].

CA9 expression was correlated with Treg numbers in 448 breast cancers: increased numbers of Treg were observed in CA9 positive tumours (median Treg = 32, n = 66) compared to CA9 negative tumours (median Treg = 10, n = 382) (Mann-Whitney U *P *< 0.001). In order to further explore whether Treg recruitment was associated with hypoxia or other factors expressed by the basal subtype, we correlated Treg numbers with CA9 expression in a subset of 327 non-basal breast cancers. CA9 positive non-basal cancers had higher numbers of Treg (median Treg = 21 per 1 mm core, n = 32) compared to CA9 negative non-basal cancers (median Treg = 8 per 1 mm core, n = 21) (Mann-Whitney U *P *= 0.044). These results suggest hypoxia promotes Treg recruitment independent of basal subtype.

### Infiltration by CXCR4 positive Treg correlates with basal phenotype and tumour hypoxia

**Figure 3:**
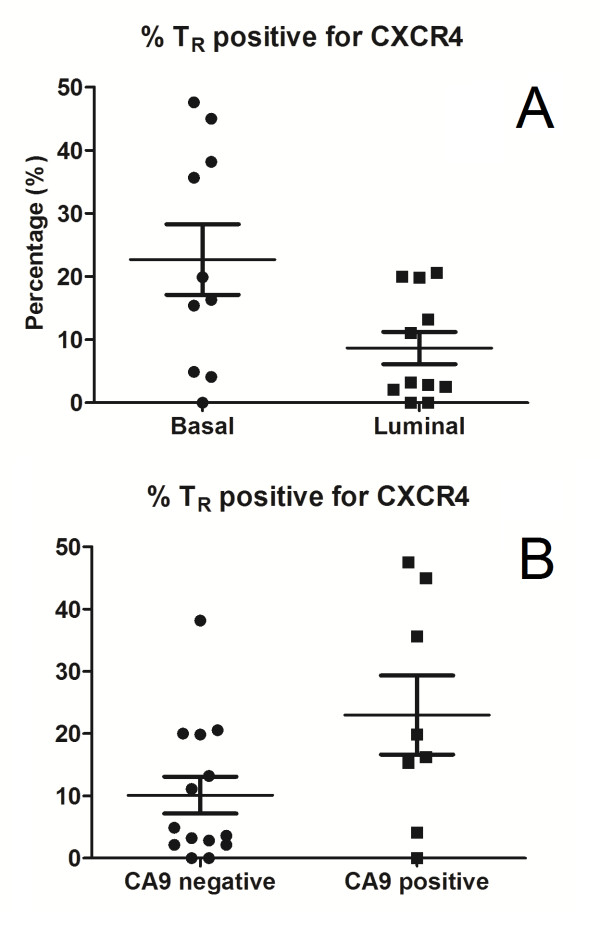
**CXCR4 expression in Treg**. **(A) **Proportion of Treg expressing CXCR4 in basal (median = 18.1%, interquartile range = 4.9% to 38.1%) vs. luminal cancers (median = 3.2%, interquartile range = 2.1% to 19.9%) (*P *= 0.029). **(B) **Proportion of Treg expressing CXCR4 in CA9 positive (median = 18.1%, interquartile range = 9.7% to 40.3%) vs. CA9 negative cancers (median = 4.3%, interquartile range = 2.1% to 19.9%) (*P *= 0.049).

Double CXCR4/FOXP3 immunoperoxidase staining was performed to evaluate CXCR4 positive Treg infiltration in whole stained sections from 10 grade 3 basal-like and 11 grade 3 luminal cancers. A higher proportion of Treg in basal-like cancers expressed CXCR (median = 18.1%, interquartile range = 4.9% to 38.1%), when compared to luminal cancers (median = 3.2%, interquartile range = 2.1% to 19.9%) (*P *= 0.029) (Figure 3a). No difference in CXCR4 positive Treg was observed between the peritumoural stroma and the tumour bed (*P *= 0.337).

Using CA9 as a surrogate marker of hypoxia, in keeping with our previous studies [32], grade 3 basal-like tumours were more likely to be hypoxic (8/10 positive for CA9), compared to grade 3 luminal cancers (0/11 positive for CA9) (*P *< 0.001). Furthermore, in hypoxic tumours with positive CA9 expression, a higher proportion of Treg was positive for CXCR4 (median = 18.1%, interquartile range = 9.7% to 40.3%) compared to CA9 negative tumours (median = 4.3%, interquartile range = 2.1% to 19.9%) (*P *= 0.049) (Figure 3b).

## Discussion

In view of the association among basal-like breast cancer, its dense lymphoid infiltrate and aggressive behaviour, we hypothesized that basal-like tumours evade the anti-tumour immune response via the recruitment of Treg. In our study, basal-like tumours were associated with high Treg and a four-fold increase in the median number of Treg compared with luminal tumours. Furthermore, this association was independent of tumour size and grade and conferred a poor survival in this subtype. These results are in keeping with our previous study in an independent cohort where Treg infiltration correlated with high tumour grade, HER2 positivity, ER negativity and poor survival [11]. and with Bohling *et al. *[45] in their comparison of 26 grade 3 triple negative cancers with 71 non-grade 3 non-triple negative cancers.

Although high Treg were also associated with an adverse outcome in luminal cancers in our cohort of HER positive patients, who were not treated with trastuzumab, accumulation of Treg did not correlate with survival. Although the reason for the latter result is unclear, one potential explanation is Treg immune suppression may have no effect on an immune response which is ineffective in the first instance. This is supported by studies where the anti-tumour immune response against HER2 cancers could be boosted by an infusion of HER2 specific T cells [46].

A number of chemokines have been implicated in the recruitment of Treg in non-neoplastic tissues [18]. Recent studies on breast cancers have shown that two of these chemokines, CCL20 [13] and CCL22 [12], may recruit Treg that express the corresponding chemokine receptors CCR6 and CCR4. Our results demonstrate CXCL12 derived from tumour cells may also be involved in Treg recruitment. While tumour CXCL12 expression correlated with tumour Treg recruitment, this did not appear to account for the increased number of Treg observed in basal-like cancers. Indeed, a lower proportion of basal-like cancers expressed CXCL12 (59%) compared with luminal cancers (74%). Our findings suggest preferential Treg recruitment in basal-like cancers may be in part explained by CXCR4 up-regulation in Treg. This is supported by *in vitro *cell migration assays where induction of CXCR4 expression in Treg resulted in their migration towards a CXCL12 gradient [19,20], and could be terminated by incubation with an anti-CXCR4 antibody [19]. Preferential accumulation of CXCR4 positive Treg, and its correlation with tumour CXCL12 expression, has also been previously demonstrated in adenocarcinomas of the lung [20] and malignant mesothelioma [21].

CXCL12 is a chemokine which exclusively binds to the CXCR4 receptor. It is also the only ligand for the CXCR4 receptor [47]. CXCR4 expression is induced by hypoxia [24,48]. Using CA9 as a surrogate marker of hypoxia [42-44], we demonstrated that hypoxia is associated with the accumulation of Treg and also the subset of CXCR4 positive Treg in breast cancer. This, together with our previous finding that hypoxia is a feature of basal-like breast cancers, suggests increased Treg infiltration in basal-like cancers may be in part due to hypoxia-induced up-regulation of CXCR4 in Treg. The correlations identified in our study are mechanistically supported by *in vitro *studies, where T cells incubated under hypoxic conditions show a time-dependent increase in HIF-1α [29-31]. Furthermore, the link between HIF-1α and CXCR4 expression is supported by a number of findings [24] including: a) reduction of CXCR4 in VHL mutated cell lines [24], b) increased IL-2 receptor (also implicated in CXCR4 up-regulation), and reduced CCR6 (another receptor implicated in Treg recruitment) expression in T cells stimulated by hypoxia [31], c) HIF-1α recruitment to the *CXCR4 *promoter in the hypoxic state [24] and d) hypoxia-induced expression of CXCR4, but not CCR6, CCR7, CXCR3 or CXCR5 [23]. HIF-1α may also induce CXCR4 expression indirectly by up-regulating the expression of FOXP3, which binds regulatory sequences upstream of the transcriptional start site of CXCR4 resulting in CXCR4 over-expression [49]. Furthermore, hypoxia increases the potency of Treg in suppressing the proliferation of effector CD4+ T cells [29,50]. There are likely also to be other cytokines regulating T cell recruitment and a comprehensive analysis of hypoxia-induced cytokines and their cognate receptors would be valuable, but need to be directed by detailed analysis of pathways regulated in these cells *in vitro*. The effect of hypoxia on Treg appears to be independent of other factors expressed by the basal subtype as the correlation between hypoxia and Treg was re-duplicated in non-basal tumours.

Loss of CXCL12 expression, in this study and in previous studies [51], is associated with a poor prognosis. Tumour cells with reduced CXCL12 in their immediate microenvironment may be at an advantage to receive endocrine CXCL12 signals, promoting their migration towards ectopic sources of the CXCR4 ligand. This is supported by mouse models where metastasis of tumour xenografts to the lung may be inhibited by endogenous CXCL12 expression in the xenografted tumour [52]. While CXCL12 expression is associated with a favourable prognosis in an analysis of all breast cancers, there may be significant heterogeneity in the impact of CXCL12 on tumour behaviour between subtypes. For example, while CXCL12 is associated with a good prognosis in non-basal breast cancers (log rank test *P *= 0.002), but no such correlation is seen for basal cancers (*P *= 0.688). In the setting of profound hypoxia and CXCR4 up-regulation in Treg, as occurs in basal-like breast cancer, CXCL12 may have a negative consequence of enhancing Treg recruitment and suppressing the anti-tumour immune response. Although CXCL12 may also recruit other T cell subsets, it appears to preferentially recruit Treg, rather than CD8 cytotoxic or CD4 helper T cells [20,53].

## Conclusions

These findings have important implications, as they suggest basal-like cancers, which are traditionally resistant to targeted therapies and may potentially respond to immunotherapy targeting Treg. Furthermore, Treg recruitment by CXCL12/CXCR4 in these cancers may potentially be modulated by treatment directed against the HIF-1α pathway. Thus there is an opportunity for clinical trials based on robust reagents directed against Treg, or antibodies blocking CXCR4, to stratify patients for anti-HIF therapies. Indeed, many potent HIF-1 inhibitors are FDA-approved cancer treatment agents including anthracyclines and topotecan enabling clinical trials to test their effectiveness [54,55]. Side effects should be minimal since these agents are chronically administered at low dose to derive their anti-HIF activity. There are some data to suggest that the anthracycline analogue, mitoxanthrone, lowers the number of Treg in tumours [56] and such agents may be combined with multiple immunotherapy strategies that also reduce Treg numbers, enabling an improved effector cell response to a vaccine.

## Abbreviations

BRCA1: breast cancer 1; early onset; CA9: carbonic anhydrase IX; CK: cytokeratin; CXCL12: chemokine (C-X-C motif) ligand 12; CXCR4: C-X-C chemokine receptor type 4; EGFR: epidermal growth factor receptor; ER: estrogen receptor; FOXP3: forkhead box P3; HER2: human epidermal growth receptor 2; HIF: hypoxia inducible factor; Treg: regulatory T cell; TMA: tissue microarray

## Competing interests

The authors declare that they have no competing interests.

## Authors' contributions

SF and MY conceived the experiments. Experiments were carried out by MY, NJ and DB. MY, SF, EM, SO, CM, GB, AH, AB and RS were involved in the collection and analysis of data. All authors were involved in writing the paper and had final approval of the submitted version.

## Supplementary Material

Additional file 1 **Supplementary Tables S1-S2**. Supplementary Table S1: **(A) **Multivariate analysis, Cox regression model, breast cancer specific survival, all tumours (n = 398). **(B) **Multivariate analysis, Cox regression model, breast cancer specific survival, patients given with hormone therapy (n = 253). Supplementary Table S2: Correlation of CXCL12 expression with clinicopathological parameters. Click here for file
