# Supplementary material for: Recruitment of regulatory T cells is correlated with hypoxia-induced CXCR4 expression, and is associated with poor prognosis in basal-like breast cancers
Source: Breast Cancer Res. 2011 Apr 26;13(2):R47. doi: 10.1186/bcr2869 (PMC3219210; doi:10.1186/bcr2869)
Supplement: Additional file 1 — Supplementary Tables S1-S2. Supplementary Table S1: (A) Multivariate analysis, Cox regression model, breast cancer specific survival, all tumours (n = 398). (B) Multivariate analysis, Cox regression model, breast cancer specific survival, patients given with hormone therapy (n = 253). Supplementary Table S2: Correlation of CXCL12 expression with clinicopathological parameters. [file bcr2869-S1.DOC]

**Supplementary Table 1a.** Multivariate analysis, Cox regression model, breast cancer specific survival, all tumors (n = 397).

|  | **Odds ratio** | **95% CI** | **p value** |
| --- | --- | --- | --- |
| **Age** | 1.01 | 0.99 – 1.03 | 0.544 |
| **Nodal status** | 3.07 | 1.42 – 6.63 | 0.004 |
| **Grade** | 1.56 | 1.03 – 2.38 | 0.038 |
| **Size > 20mm** | 2.52 | 1.57 – 4.04 | < 0.001 |
| **ER** | 0.55 | 0.35 – 0.86 | 0.009 |
| **HER2** | 1.51 | 0.90 – 2.53 | 0.116 |
| **Hormonal therapy** | 0.64 | 0.40 – 1.03 | 0.068 |
| **Chemotherapy** | 0.97 | 0.46 – 2.06 | 0.941 |
| **T_reg_ ≥ 15** | 1.62 | 1.02 – 2.55 | 0.040 |

ER: estrogen receptor; Treg: regulatory T cells

**Supplementary Table 1b.** Multivariate analysis, Cox regression model, breast cancer specific survival, patients given with hormone therapy (n = 253).

|  | **Odds ratio** | **95% CI** | **p value** |
| --- | --- | --- | --- |
| **Age** | 1.01 | 0.60 – 2.03 | 0.744 |
| **Nodal status** | 3.10 | 2.08 – 7.64 | 0.006 |
| **Grade** | 2.01 | 1.12 – 3.62 | 0.020 |
| **Size > 20mm** | 3.99 | 1.27 – 4.18 | < 0.001 |
| **ER** | 0.57 | 0.33 – 0.99 | 0.047 |
| **HER2** | 1.51 | 0.88 – 3.09 | 0.116 |
| **T_reg_ ≥ 15** | 1.78 | 1.01 – 3.15 | 0.040 |

ER: estrogen receptor; Treg: regulatory T cells

**Supplementary Table 2.** Correlation of CXCL12 expression with clinicopathological parameters.

|  | **Negative CXCL12** | **Positive CXCL12** | **p value** |
| --- | --- | --- | --- |
| **Tumor size (mm)**  Median | 22.1 | 20.9 | 0.497 |
| **Tumor grade**  1  2  3 | 9 (11%)  25 (32%)  45 (57%) | 29 (18%)  59 (38%)  69 (44%) | 0.135 |
| **Nodal status**  Negative  Positive | 44 (56%)  35 (44%) | 83 (54%)  72 (46%) | 0.755 |
| **ER**  Negative  Positive | 30 (38%)  49 (62%) | 39 (25%)  116 (75%) | 0.076 |
| **HER2**  Negative  Positive | 53 (71%)  22 (29%) | 130 (84%)  25 (16%) | 0.015 |
| **Endocrine Rx**  Negative  Positive | 40 (51%)  39 (49%) | 71 (45%)  86 (55%) | 0.432 |
| **Chemotherapy**  Negative  Positive | 44 (56%)  35 (44%) | 97 (62%)  60 (38%) | 0.368 |

ER: estrogen receptor; Rx: therapy; Treg: regulatory T cells
